# Current Innovations in Peripheral Nerve Stimulation

**DOI:** 10.1155/2018/9091216

**Published:** 2018-09-13

**Authors:** Raghavendra Nayak, Ratan K. Banik

**Affiliations:** Department of Anesthesiology, School of Medicine, University of Minnesota, Minneapolis, MN, USA

## Abstract

Peripheral nerve stimulation has been used in the treatment of several chronic pain conditions including pain due to peripheral nerve dysfunctions, complex regional pain syndrome, and cranial neuralgias. It has been shown to be effective for chronic, intractable pain that is refractory to conventional therapies such as physical therapy, medications, transcutaneous electrical stimulations, and nerve blocks. Recently, a new generation of peripheral nerve stimulation devices has been developed; these allow external pulse generators to transmit impulses wirelessly to the implanted electrode, and their implantation is significantly less invasive. In this review, we discuss the history, pathophysiology, indications, implantation process, and outcomes of employing peripheral nerve stimulation to treat chronic pain conditions.

## 1. History and Introduction

The use of electricity for treatment of chronic pain can be traced back to the Romans, who used electric eels, a fresh water predator that can generate and release electricity to stun prey, for the treatment of various painful conditions. The modern era of neuromodulation for intractable and chronic pain was conceptualized after the publication of Wall and Melzack's gate control theory of pain in 1965 [1]. The first report of pain relief following electrical stimulation comes from Wall and Sweet, in 1967 [2]. Electrical stimulation was applied to eight patients with chronic neuropathic pain. The stimulation consisted of 0.1ms pulses at a rate of 100 Hz for two minutes; the voltage was adjusted until patients reported a tingling sensation in the affected area. After completion of the procedure, four of the eight patients experienced more than half an hour of pain relief. Since then, there have been tremendous advancements in the field of neuromodulation, including peripheral nerve stimulation (PNS). These advancements include improvement in ultrasound technologies, integration of ultrasound into clinical practice, percutaneous implantation techniques, smaller devices, and rechargeable and larger-capacity batteries. Within the last decade, PNS has been shown to be effective in the treatment of several painful conditions including complex regional pain syndrome [3, 4], postherpetic neuralgia, posttraumatic neuropathy [5], cranial neuralgias [6], and various headache disorders [6].

## 2. Pathophysiology

The most attractive hypothesis for the mechanism underlying PNS is gate control theory, which was published in* Science* in 1965 [1]. Gate control theory suggests that “gates” at the spinal cord dorsal horn laminae competitively regulate nonnociceptive and nociceptive input. Nonnociceptive input activates large-diameter sensory fibers that close the “gates” to nociceptive input, which is otherwise carried via small-diameter fibers. The input from small-diameter fibers opens the “gates” and results in nociception (pain). This means that* pain* is perceived when these physiological ‘gates' give way to* pain* signals and is less intense or not perceived when the gate closes. The theory explains why* rubbing* or massaging a* painful* site relieves pain: it causes stimulation of nonnociceptive fibers, which close the ‘gates' [7]. Subsequently, techniques for peripheral nerve stimulation [8] were developed on the same principle.

Additional theories that have attempted to explain the pain relief provided by PNS include (1) excitation failure in c-fiber nociceptors and suppression of dorsal horn activity, (2) stimulation-induced blockade of cell membrane depolarization preventing axon conduction propagation, (3) decreased hyperexcitability and long-term potentiation of dorsal horn neurons, and (4) depletion of excitatory amino acids (glutamate, aspartate) and increased release of inhibitory transmitters (GABA) [9].

It should be noted, however, that pain perception is a product of the brain's processing of afferent inputs; the perception of pain involves numerous sensory, affective, and cognitive components [10]. Furthermore, afferent pathways interact with each other in many ways other than the “gates” mentioned above. Ultimately, it is the brain that controls the perception of pain and determines which stimuli are useful and which are to be ignored [11].

## 3. Indications and Patient Selection

PNS has been shown to be efficacious in several chronic pain conditions including trigeminal neuropathic pain [12–16], episodic cluster headache (supraorbital nerve stimulation) [17, 18], chronic migraine/headache disorders (occipital nerve stimulation) [19, 20], fibromyalgia (C2 area stimulation) [21, 22], postherpetic neuralgia [23–26], complex regional pain syndrome type I [4, 27] and type II [28], isolated peripheral neuropathy [29], ilioinguinal, iliohypogastric, and lateral femoral cutaneous neuralgia [30], back pain [31], foot pain (tibial nerve stimulation) [32], and coccydynia [21, 33].

In general, PNS is recommended when a patient's symptoms are refractory to conventional interventions such as physical therapy, medications, transcutaneous electrical nerve stimulation (TENS), and nerve blocks. Prior to considering PNS, correctable pathologies such as nerve entrapment should be excluded with diagnostic and imaging studies. Additionally, psychological evaluation should be undertaken to identify factors that may impact the outcome, such as secondary gain, mood, and personality disorders.

The criteria for patient selection are as follows:

1. Pain consistent with the sensory distribution of a single peripheral nerve
2. A positive diagnostic peripheral nerve block
3. Exclusion of nerve entrapment neuropathies
4. The patient is free of major psychological or psychiatric disease

These selection criteria are a consensus based on the clinical experience and suggestions of multiple published studies. However, no published studies show any predictive value for diagnostic peripheral nerve blocks or the efficacy of a TENS unit on PNS success [34]. Contraindications for the use of PNS mainly relate to surgical risk and include (1) coagulopathy, (2) infection in the surgical site, (3) psychiatric illness, (4) a failed diagnostic trial, (5) requirement of periodic MRIs, such as for cancer patients, and (6) complete sensory loss.

Postoperative complications include infection, nerve damage, electrode migration, mechanical failure including disconnection of hardware, failure to provide adequate stimulation, and pain relief, pain, and cosmetic concerns.

## 4. Implantation Process

An implantable peripheral nerve stimulator consists of a single electrode with 8-16 contact leads. The electrode is implanted in close proximity to the targeted peripheral nerve, and either an implanted or external battery is used to stimulate the electrode. Traditionally, the placement of a PNS is a two-stage procedure. The first stage or trial phase involves the temporary implantation of an electrode to assess symptomatic relief. Most commonly, the electrode is placed through a 14G Tuoy needle in close proximity to the target nerve. Feletti and colleagues used small 3 mm incisions in the supra-auricular or preauricular area to access V2 and V3 divisions of the trigeminal nerve [12]. They observed better pain relief when electrodes were placed at the area of hyperalgesia compared with allodynia along the distribution of the trigeminal nerve. It is possible that, with this method, the electrodes preferentially stimulate A-beta fibers, which close the ‘gates' in the spinal cord dorsal horn cells.

After placement of the electrode, it is sutured to adjacent myofascial structures in order to minimize migration risk. An extension cable is connected and tunneled to an external, temporary power source. Over the next five to seven days, the patient is allowed to test the system. If the symptomatic relief is adequate, the patient proceeds to the second stage and receives a permanent implantation. This procedure is similar to the trial, except a subcutaneous pocket is created to house the battery and programming component.

The use of ultrasound technology during implantation allows for percutaneous placement of the PNS electrode [25] and almost eliminates the need for skin incision and tissue dissection. Under ultrasound guidance, a 14-gauge needle is used to pierce the cutaneous tissues and visualize the target nerve and/or its fascicles. Once the needle approaches the target area, an electrode is passed through it into the vicinity of the nerve. Using this approach, multiple electrodes may be implanted in order to account for variability in the position of the nerve during normal movements. Permanent implantation of electrodes can similarly be performed under ultrasound guidance. The electrode is connected to an implantable pulse generator, which is placed next to the stimulated area in a separate pocket, typically in the abdomen, chest wall, buttock, or thigh.

## 5. Current Innovations

**Figure 1:**
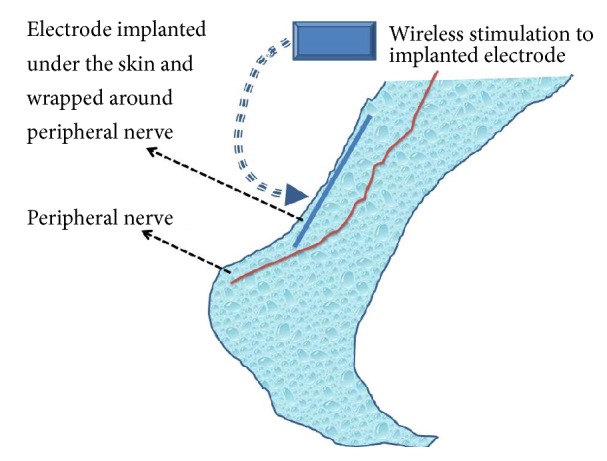
Schematic presentation of wireless peripheral nerve stimulation. An extremely small, insulated electrical lead is inserted adjacent to peripheral nerve through a 14G Tuoy needle under ultrasound guidance [25, 35]. The lead insertion is similar to perineural catheter placement. No additional equipment is required to be inserted into patient's body. The procedure is rapid and relatively less traumatic. The external pulse generators, which are wearable in belts, fabric, jewelry, etc., provide power to the implanted wireless leads. The technique eliminates need for an implantable pulse generator (IPG) and tunneling of the electrodes to IPG, thereby reducing the expense of these therapies. The electrical energy is transmitted from external pulse generator to the implanted leads, inducing action potentials in nearby neurons.

As interest in peripheral nerve stimulators increases, so do research and innovation in corresponding hardware. Recently, a new generation of devices has been developed that allows for external pulse generators to transmit impulses wirelessly to the implanted electrode, produced by Stimwave, Bioness, and SPR Therapeutics (Figure 1).

With this new technology, an extremely small, insulated electrical lead is inserted adjacent to peripheral nerve through a 14G Tuoy needle and under ultrasound guidance (Figure 1) [25, 35]. This insertion is similar to perineural catheter placement, which is widely utilized in regional anesthesia and acute pain medicine. The procedure is rapid and relatively less traumatic than conventional implantation. Power is provided to the implanted wireless lead by external pulse generators that are wearable in belts, fabric, jewelry, etc. This technique eliminates the need for an implantable pulse generator (IPG) and tunneling of the electrodes to the IPG, thereby reducing the expense and morbidities. The use of this technology has been evaluated in a study of acute postoperative pain from total knee arthroplasty [36]. Ilfeld et al. inserted percutaneous peripheral nerve electrodes using ultrasound guidance in close proximity to the femoral and/or sciatic nerve(s). With the delivery of current, pain decreased an average of 63% at rest, with four of the five subjects having relief > 50%. The study was funded by SPR Therapeutics.

In agreement with those results, Deer et al. demonstrated in a prospective, multicenter, randomized, double-blinded, partial crossover study that a peripheral nerve stimulator with an external power source provided significant relief compared to control in patients with severe intractable chronic pain of peripheral nerve origin [37–40]. In this study, 94 patients were implanted and then randomized to the treatment (N=45) or the control group (N=49). The patients who received active stimulation achieved a statistically significant higher response rate of 38% versus the 10% found in the control group (*p* < 0.0048). The mean pain reduction was 27.2% from baseline compared to a 2.3% reduction in the control group (*p* < 0.0001). During the partial crossover period, the treatment group again demonstrated statistically significant improvement in pain relief. This study was funded by Bioness Inc., manufacturer of the StimRouter.

## 6. Long-Term Outcomes

There is little evidence regarding the long-term efficacy of PNS therapy in managing chronic pain. Case reports and retrospective reviews do support that PNS may be helpful in chronic pain from peripheral nerve injuries. Mobbs et al. reviewed 38 patients, classifying them into four groups based on pain type: blunt or sharp nerve trauma (14/38), iatrogenic injuries from surgery (9/38), inadvertent injection of a nerve (9/38), and postsurgery for entrapment or tumor (8/38) [41]. Following the implantation of peripheral nerve stimulators, over 60% of these patients had significant improvement in their symptoms and lifestyle quality. A total of 15 patients reported fair or poor results (39%). Six patients required removal of their stimulators (15%) due to infection or reduction of pain control. Forty-seven percent (18/38) reported improvement in their activity levels.

In a separate study, Slavin et al. reviewed the records of ten patients who had intractable occipital neuralgia treated with peripheral nerve stimulators [42, 43]. Seven of these patients demonstrated beneficial effects (good pain control, employment, and decrease in oral pain medication intake) that lasted for 5-32 months (mean 22 months).

Verill et al. performed a prospective observational study in a private practice setting, consisting of 100 patients who received PNS for the treatment of chronic craniofacial, thorax, lumbosacral, abdominal, pelvic, and groin pain conditions [44]. They observed an average pain reduction of 4.2 ± 2.5 points on an 11-point scale (baseline pain score 7.4 ± 1.7 decreased to 3.2 ± 2.3 in follow-ups). At a follow-up period of 8.1 ± 4.7 months (range 1–23 months) after PNS implantation, an overall 72% of patients reduced their analgesic use.

## 7. Conclusion

In context of the current opioid epidemic, when opioids kill nearly 42,000 people each year [45], it is important for healthcare providers to recognize potential nondrug therapies for treating chronic pain. Although randomized controlled studies are currently lacking, there is hope that neuromodulation devices may bring a new horizon for the treatment of chronic pain related to trauma, nerve injuries, and stroke. Recent significant advances in the miniaturization of neuromodulation devices and an ultrasound-guided, minimally invasive implantation technique may be a tremendous step forward in nondrug solutions for chronic pain.

## 8. High Yield Points

- As techniques, including ultrasound guidance, evolve along with the technology of PNS systems, the utilization of this technology for the treatment of intractable chronic neuropathic pain and acute postoperative pain will likely increase.
- Currently, most data regarding the efficacy of PNS is retrospective and/or observational. There are several RCTs currently underway evaluating the efficacy in PNS in treating intractable pain conditions.
- The mechanism underlying the analgesic effects of PNS is thought to relate to the gate control theory of pain. Research is needed to explore other mechanisms that may contribute.
- When evaluating for PNS, rigorous patient selection criteria should be followed and the exclusion of treatable conditions is paramount. In addition, one should confirm that all other treatment options have been exhausted.

## 9. Questions for CME Activity

1. What is a common indication for implantation of a peripheral nerve stimulator?
  - Chronic Myofascial Pain
  - Fibromyalgia
  - Occipital neuralgia
  - Migraine Headaches
2. Which of the following patients would be a good candidate for PNS?
  - 60 year old with carpal tunnel syndrome
  - 55 year old with painful diabetic neuropathy
  - 40 year old with fibromyalgia
  - 50 year old with type II CPRS
3. Which of the following is not a part of the patient selection criteria in order to consider a PNS trial?
  - EMG confirmation of a diffuse peripheral neuropathy
  - Diagnostic peripheral nerve block
  - Lack of entrapment neuropathies
  - Pain predominantly in a single peripheral nerve distribution

Answers: 1C, 2D, and 3A.

## References

[1] Melzack R., Wall P. D. (1965). Pain mechanisms: a new theory. *Science*.

[2] Wall P. D., Swert W. H. (1967). Temporary abolition of pain in man. *Science*.

[3] Goroszeniuk T., Pang D., Shetty A., Eldabe S., O'Keeffe D., Racz G. (2014). Percutaneous peripheral neuromodulation lead insertion using a novel stimulating coudé needle. *Neuromodulation: Technology at the Neural Interface*.

[4] Racz G. B., Browne T., Lewis R. (1988). Peripheral stimulator implant for treatment of causalgia caused by electrical burns.. *Texas Medicine*.

[5] Law J. D., Swett J., Kirsch W. M. (1980). Retrospective analysis of 22 patients with chronic pain treated by peripheral nerve stimulation. *Journal of Neurosurgery*.

[6] Slavin K. V., Colpan M. E., Munawar N., Wess C., Nersesyan H. (2006). Trigeminal and occipital peripheral nerve stimulation for craniofacial pain: a single-institution experience and review of the literature.. *Neurosurgical Focus*.

[7] Gammon G. D., Starr I. (1941). Studies on the Relief of Pain by Counterirritation. *The Journal of Clinical Investigation*.

[8] Tashani O., Johnson M. I. (2009). Transcutaneous electrical nerve stimulation (TENS) a possible aid for pain relief in developing countries?. *Libyan Journal of Medicine*.

[9] Fishman S., Ballantyne J., Rathmell J. (2010). *Bonica's management of pain Wolters Kluwer Health/Lippincott*.

[10] National Research Council (US) Committee on Recognition and Alleviation of Pain in Laboratory Animals (2009). *Recognition and Alleviation of Pain in Laboratory Animals*.

[11] Tracey I., Mantyh P. W. (2007). The cerebral signature for pain perception and its modulation. *Neuron*.

[12] Feletti A., Santi G. Z., Sammartino F., Bevilacqua M., Cisotto P., Longatti P. (2013). Peripheral trigeminal nerve field stimulation: Report of 6 cases. *Neurosurgical Focus*.

[13] Klein J., Sandi-Gahun S., Schackert G., Juratli T. A. (2015). The impact of peripheral nerve field stimulation on treatment of facial pain syndromes including trigeminal neuropathy attributed to multiple sclerosis. *Multiple Sclerosis Journal*.

[14] Klein J., Sandi-Gahun S., Schackert G., Juratli TA. (2015). Peripheral nerve field stimulation for trigeminal neuropathic pain syndromes and persistent idiopathic facial pain. *Cephalalgia*.

[15] Klein J., Sandi-Gahun S., Schackert G., Juratli T. A. (2015). Peripheral nerve field stimulation for trigeminal neuralgia, trigeminal neuropathic pain, and persistent idiopathic facial pain. *Cephalalgia*.

[16] Lenchig S., Cohen J., Patin D. (2012). A minimally invasive surgical technique for the treatment of posttraumatic trigeminal neuropathic pain with peripheral nerve stimulation. *Pain Physician*.

[17] Asensio-Samper J. M., Villanueva V. L., Pérez A. V. (2008). Peripheral neurostimulation in supraorbital neuralgia refractory to conventional therapy. *Pain Practice*.

[18] Narouze S. N., Kapural L. (2007). Supraorbital nerve electric stimulation for the treatment of intractable chronic cluster headache: a case report. *Headache: The Journal of Head and Face Pain*.

[19] Matharu M. S., Bartsch T., Ward N., Frackowiak R. S. J., Weiner R., Goadsby P. J. (2004). Central neuromodulation in chronic migraine patients with suboccipital stimulators: a PET study. *Brain*.

[20] Melvin E. A., Jordan F. R., Weiner R. L., Primm D. (2007). Using peripheral stimulation to reduce the pain of C2-mediated occipital headaches: A preliminary report. *Pain Physician*.

[21] Slavin K. V. (2007). Peripheral neurostimulation in fibromyalgia: A new frontier?!. *Pain Medicine*.

[22] Thimineur M., De Ridder D. (2007). C2 area neurostimulation: A surgical treatment for fibromyalgia. *Pain Medicine*.

[23] Dunteman E. (2002). Peripheral nerve stimulation for unremitting ophthalmic postherpetic neuralgia. *Neuromodulation: Technology at the Neural Interface*.

[24] Johnson M. D., Burchiel K. J. (2004). Peripheral stimulation for treatment of trigeminal postherpetic neuralgia and trigeminal posttraumatic neuropathic pain: A pilot study. *Neurosurgery*.

[25] Lerman I. R., Chen J. L., Hiller D. (2015). Novel High-Frequency Peripheral Nerve Stimulator Treatment of Refractory Postherpetic Neuralgia: A Brief Technical Note. *Neuromodulation: Technology at the Neural Interface*.

[26] Upadhyay S. P., Rana S. P., Mishra S., Bhatnagar S. (2010). Successful treatment of an intractable postherpetic neuralgia (PHN) using peripheral nerve field stimulation (PNFS). *American Journal of Hospice and Palliative Medicine*.

[27] Hassenbusch S. J., Stanton-Hicks M., Schoppa D., Walsh J. G., Covington E. C. (1996). Long-term results of peripheral nerve stimulation for reflex sympathetic dystrophy. *Journal of Neurosurgery*.

[28] Jeon I.-C., Kim M.-S., Kim S.-H. (2009). Median nerve stimulation in a patient with complex regional pain syndrome type II. *Journal of Korean Neurosurgical Society*.

[29] Eisenberg E., Waisbrod H., Gerbershagen H. U. (2004). Long-Term Peripheral Nerve Stimulation for Painful Nerve Injuries. *The Clinical Journal of Pain*.

[30] Stinson L. W., Roderer G. T., Cross N. E., Davis B. E. (2001). Peripheral subcutaneous electrostimulation for control of intractable post-operative inguinal pain: A case report series. *Neuromodulation: Technology at the Neural Interface*.

[31] Kapural L., Gilmore C. A., Chae J. (2018). Percutaneous Peripheral Nerve Stimulation for the Treatment of Chronic Low Back Pain: Two Clinical Case Reports of Sustained Pain Relief. *Pain Practice*.

[32] Chan I., Brown A. R., Park K., Winfree C. J. (2010). Ultrasound-guided, percutaneous peripheral nerve stimulation: Technical note. *Neurosurgery*.

[33] Granville M., Brennan P., Jacobson R. E. (2017). Bilateral Peripheral Nerve Field Stimulation for Intractable Coccygeal Pain: A Case Study Using Dual Lead Intercommunication. *Cureus*.

[34] Slavin K. V. (2008). Peripheral Nerve Stimulation for Neuropathic Pain. *Neurotherapeutics*.

[35] Billet B., Wynendaele R., Vanquathem N. E. (2018). A Novel Minimally Invasive Wireless Technology for Neuromodulation via Percutaneous Intercostal Nerve Stimulation for Post-Herpetic Neuralgia: A Case Report with Short-Term Follow-up. *Pain Practice*.

[36] Ilfeld B. M., Grant S. A., Gilmore C. A. (2017). Neurostimulation for Postsurgical Analgesia: A Novel System Enabling Ultrasound-Guided Percutaneous Peripheral Nerve Stimulation. *Pain Practice*.

[37] Alo K. M. (2016). Prospective, Multicenter, Randomized, Double-Blinded, Partial Crossover Study to Assess the Safety and Efficacy of the Novel Neuromodulation System in the Treatment of Patients With Chronic Pain of Peripheral Nerve Origin COMMENT. *Neuromodulation*.

[38] Deer T., Pope J., Benyamin R. (2016). Prospective, multicenter, randomized, double-blinded, partial crossover study to assess the safety and efficacy of the novel neuromodulation system in the treatment of patients with chronic pain of peripheral nerve origin. *Neuromodulation: Technology at the Neural Interface*.

[39] Weiner R. (2016). Prospective, Multicenter, Randomized, Double-Blinded, Partial Crossover Study to Assess the Safety and Efficacy of the Novel Neuromodulation System in the Treatment of Patients With Chronic Pain of Peripheral Nerve Origin COMMENT. *Neuromodulation*.

[40] Yearwood T., Multicenter Prospective., Double-Blinded Randomized. (2016). Prospective, Multicenter, Randomized, Double-Blinded, Partial Crossover Study to Assess the Safety and Efficacy of the Novel Neuromodulation System in the Treatment of Patients With Chronic Pain of Peripheral Nerve Origin COMMENT. *Neuromodulation*.

[41] Mobbs R. J., Nair S., Blum P. (2007). Peripheral nerve stimulation for the treatment of chronic pain. *Journal of Clinical Neuroscience*.

[42] Sagher O., Patil P. G., Lozano A. M., Machado A., Rezai A. R. (2006). Peripheral neurostimulation for treatment of intractable occipital neuralgia: Commentary. *Neurosurgery*.

[43] Slavin K. V., Nersesyan H., Wess C. (2006). Peripheral neurostimulation for treatment of intractable occipital neuralgia. *Neurosurgery*.

[44] Verrills P., Vivian D., Mitchell B., Barnard A. (2011). Peripheral Nerve Field Stimulation for Chronic Pain: 100 Cases and Review of the Literature. *Pain Medicine*.

[45] Seth P., Scholl L., Rudd R. A., Bacon S. (2018). overdose deaths involving opioids, cocaine, and psychostimulants - United States, 2015-2016. *Morbidity and Mortality Weekly Report*.

